# Relevance of neuroimaging for neurocognitive and behavioral outcome after pediatric traumatic brain injury

**DOI:** 10.1007/s11682-017-9673-3

**Published:** 2017-01-14

**Authors:** Marsh Königs, Petra JW Pouwels, LW Ernest van Heurn, Roel Bakx, R Jeroen Vermeulen, J Carel Goslings, Bwee Tien Poll-The, Marleen van der Wees, Coriene E Catsman-Berrevoets, Jaap Oosterlaan

**Affiliations:** 1 Clinical Neuropsychology Section, FGB VU University Amsterdam, Van der Boechorststraat 1, 1081BT Amsterdam, The Netherlands; 2 Emma Children’s Hospital, Academic Medical Center, Amsterdam, The Netherlands; 3 Department of Physics and Medical Technology, VU University Medical Center, Amsterdam, The Netherlands; 4 Neuroscience Campus Amsterdam, Amsterdam, The Netherlands; 5 Pediatric Surgical Center of Amsterdam, Emma Children’s Hospital Academic Medical Center and VU University Medical Center, Amsterdam, The Netherlands; 6 Department of Pediatric Neurology, VU University Medical Center, Amsterdam, The Netherlands; 7 Department of Pediatric Neurology, Maastricht University, Medical Center, Maastricht, The Netherlands; 8 Trauma Unit, Academic Medical Center, Amsterdam, The Netherlands; 9 Department of Pediatric Neurology, Emma Children’s Hospital Academic Medical Centre, Amsterdam, The Netherlands; 10 Libra Rehabilitation Medicine and Audiology, ‘Blixembosch’, Eindhoven, The Netherlands; 11 Department of Pediatric Neurology, Erasmus University Hospital/ Sophia Children’s Hospital, Rotterdam, The Netherlands; 12 Department of Pediatrics, VU University Medical Center, Amsterdam, The Netherlands

**Keywords:** Diffusion tensor imaging, Tract-based spatial statistics, Pediatrics, Traumatic brain injury, Neurocognitive functioning, Behavior problems

## Abstract

This study aims to (1) investigate the neuropathology of mild to severe pediatric TBI and (2) elucidate the predictive value of conventional and innovative neuroimaging for functional outcome. Children aged 8–14 years with trauma control (TC) injury (*n* = 27) were compared to children with mild TBI and risk factors for complicated TBI (mild^RF+^, *n* = 20) or moderate/severe TBI (*n* = 17) at 2.8 years post-injury. Neuroimaging measures included: acute computed tomography (CT), volumetric analysis on post-acute conventional T1-weighted magnetic resonance imaging (MRI) and post-acute diffusion tensor imaging (DTI, analyzed using tract-based spatial statistics and voxel-wise regression). Functional outcome was measured using Common Data Elements for neurocognitive and behavioral functioning. The results show that intracranial pathology on acute CT-scans was more prevalent after moderate/severe TBI (65%) than after mild^RF+^ TBI (35%; *p* = .035), while both groups had decreased white matter volume on conventional MRI (*p*s ≤ .029, *d*s ≥ −0.74). The moderate/severe TBI group further showed decreased fractional anisotropy (FA) in a widespread cluster affecting all white matter tracts, in which regional associations with neurocognitive functioning were observed (FSIQ, Digit Span and RAVLT Encoding) that consistently involved the corpus callosum. FA had superior predictive value for functional outcome (i.e. intelligence, attention and working memory, encoding in verbal memory and internalizing problems) relative to acute CT-scanning (i.e. internalizing problems) and conventional MRI (no predictive value). We conclude that children with mild^RF+^ TBI and moderate/severe TBI are at risk of persistent white matter abnormality. Furthermore, DTI has superior predictive value for neurocognitive out-come relative to conventional neuroimaging.

**Electronic supplementary material:**

The online version of this article (doi:10.1007/s11682-017-9673-3) contains supplementary material, which is available to authorized users.

## Introduction

Traumatic brain injury (TBI) is the leading cause of disability in children and young adults (World Health Organization 2006). Children with moderate to severe TBI are typically at risk of poor functional outcome in terms of neurocognitive impairment and behavior problems. Neurocognitive impairments include deficits in attention and working memory, learning and memory, and executive functioning (Babikian and Asarnow 2009), whereas behavior problems include anxiety, depression and aggression (Li and Liu 2013). Recent evidence indicates that even after mild TBI, children with risk factors for intracranial pathology (i.e. mild^RF+^; e.g. skull fracture, persistent vomiting, focal neurological impairment) are at risk of poor neurocognitive and behavioral outcome (Königs et al. 2015a, b). Importantly, functional outcome of mild to severe TBI is characterized by a distinct inter-individual heterogeneity that remains poorly understood (Polinder et al. 2015), and therefore complicates reliable prognosis of neurocognitive and behavioral outcome. The complexity of TBI neuropathology is thought to represent a crucial source of heterogeneity in functional outcome (Bigler et al. 2013a). Therefore, a better understanding of the relations between both conventional and innovative neuroimaging and functional outcome may contribute to a more reliable prognosis for children with TBI.

While the neuropathology of mild TBI remains obscure (Bigler and Maxwell 2012), the literature shows that moderate to severe TBI involves complex interactions between primary and secondary injury mechanisms, involving focal injuries (i.e. contusions and hemorrhages), diffuse injuries (i.e. diffuse axonal injury) as well as subsequently raised intracranial pressure and neurotoxic biochemical cascades (Bigler et al. 2013). Recent evidence suggests that the widespread and persistent disruption of white matter integrity observed after pediatric TBI (Roberts et al. 2014) plays a pivotal role in functional outcome, through the disturbance of neural networks that give rise to neurocognitive and behavioral functioning (Sharp et al. 2014). Unfortunately, clinical neuroimaging modalities (i.e. computed tomography [CT] and conventional magnetic resonance imaging [MRI], e.g. T1-weighted imaging) have limited sensitivity for the diffuse impact of TBI on white matter integrity (Mittl et al. 1994; Sigmund et al. 2007). This suggests that clinical neuroimaging modalities may lack the potential to account for the complexity of TBI neuropathology in the prognosis for functional outcome. In line with this idea, multiple studies have questioned the prognostic value of CT-scans and conventional MRI for functional outcome of pediatric TBI (Blackman et al. 2003; Gerlach et al. 2009; Mendelsohn et al. 1992).

Diffusion tensor imaging (DTI) is an innovative MRI technique with superior sensitivity for detecting the impact of TBI on white matter integrity as compared to CT and conventional MRI (Mac Donald et al. 2011; Niogi and Mukherjee 2010). Meta-analytic evidence also supports the predictive value of DTI parameters for neurocognitive functioning in the chronic phase of recovery from pediatric TBI (Roberts et al. 2016). Indeed, a convincing body of recent studies consistently reported associations between DTI parameters and multiple aspects of neurocognitive functioning, including attention and working memory (Ewing-Cobbs et al. 2008; Levin et al. 2008; Treble et al. 2013; Van Beek et al. 2015; Wilde et al. 2011; Wozniak et al. 2007; Wu et al. 2010), learning and memory (Dennis et al. 2015; Mccauley et al. 2011) and executive functioning (Dennis et al. 2015; Wilde et al. 2006; Wozniak et al. 2007; Wu et al. 2010). Although the literature on behavioral functioning is sparse, there is also some evidence supporting the relation between DTI and behavior problems after pediatric TBI (Johnson et al. 2011; Max et al. 2012) and the predictive value of DTI for the development of post-injury psychiatric disorders (Max et al. 2012).

Despite convincing evidence supporting the relevance of DTI for functional outcome after TBI, only one study compared DTI to conventional neuroimaging in terms of its predictive value (Oni et al. 2010). This study reported that DTI has superior predictive value for global functional outcome (i.e. Glasgow Outcome Scale) as compared to CT and/or conventional MRI. A meta-analytic review further revealed that the available literature on the predictive value of DTI is limited to region-of-interest (ROI) analyses (Roberts et al. 2016). The selection of ROIs greatly differs between studies, is prone to investigator bias and may provide incomplete information about regional associations between white matter integrity and aspects of functional outcome (Poldrack 2007; Roberts et al. 2016). Furthermore, the available studies have used a wide range of heterogeneous instruments to measure neurocognitive functioning, complicating an integrative interpretation of results across the literature (Roberts et al. 2016).

The current study aims to (1) investigate the neuropathology of mild^RF+^ to severe pediatric TBI and (2) elucidate the predictive value of conventional and innovative neuroimaging (clinical evaluation of acute CT scans, volumetric analysis of post-acute T1-weighted MRI scans and tract-based spatial statistics [TBSS] on post-acute DTI scans) for functional outcome. Functional outcome was defined in terms of neurocognitive functioning (i.e. intelligence, attention and working memory, verbal learning and memory) and behavioral functioning (i.e. parent and teacher ratings of internalizing and externalizing problems) as measured using Common Data Elements to maximize utility of the results for clinical and research purposes (McCauley et al. 2012). TBSS is a data-driven, model-free alternative to the ROI approach in DTI analysis, which was used to identify the white matter tracts that are affected by TBI. Pearson correlations and voxel-wise regression were subsequently used to investigate the predictive value of neuroimaging parameters for neurocognitive and behavioral outcome. Based on the available evidence, we expected that DTI parameters would have superior predictive value for functional outcome after pediatric TBI as compared to neuroimaging parameters from conventional neuro-imaging. The results of our study may contribute to the prognosis of neurocognitive and behavioral functioning after pediatric TBI, facilitating early planning of rehabilitation services and management of family expectations in clinical practice.

## Methods

### Participants

This study compared a group of 37 children with TBI to a group of 27 children with trauma control (TC) injury not involving the head, to control for pre-injury risk factors of traumatic injury and psychological effects of hospitalization (Max et al. 1998). Data were collected as part of a follow-up on a consecutive cohort that was retrospectively recruited from three university-affiliated level I trauma centers and three rehabilitation centers in the Netherlands (Königs et al. 2015a). Inclusion criteria were: (1) age 8–14 years at time of follow-up; (2) proficient in the Dutch language; (3) children in the TBI group were required to have a history of hospital admission with a clinical diagnosis of either: (a) mild TBI (GCS = 15–13, loss of consciousness [LOC] duration ≤30 min, post-traumatic amnesia [PTA] duration ≤1 h) with at least one of the following risk factors for complicated TBI (mild^RF+^ TBI) according to the European Federation of Neurological Societies guidelines on mild TBI: impaired consciousness (GCS = 13–14), focal neurological deficits, persistent vomiting (≥3 episodes), post-injury epileptic seizure, progressive headache and abnormal head CT-scan (Vos and Battistin 2002); or (b) moderate/severe TBI (GCS = 12–3, LOC duration >30 min, PTA duration >1 h (Teasdale and Jennett 1976)); and (4) children in the TC group were required to have a history of hospital admission for traumatic injuries below the clavicles (American College of Surgeons 2004). Exclusion criteria were: (1) previous TBI; (2) visual disorder interfering with neurocognitive testing; or (3) current neurological condition with known effects on neurocognitive functioning, other than TBI, as documented in medical records or reported in a parent-questionnaire for premorbid functioning.

### Background information

Data on gender, age, socio-economic status (SES) and diagnosed psychiatric or learning disorders were collected using a parental questionnaire. SES was defined as the average level of parental education ranging from 1 (no education) to 8 (postdoctoral education; Statistics Netherlands 2006).

### Injury severity

Diagnosed injuries, the lowest score on the GCS on the day of admission, presence of the described risk factors for complicated mild TBI (Vos and Battistin 2002), and length of hospital stay were extracted from medical files, as was information on any executed surgical procedures.

### CT

CT-scans were performed as part of the protocol for acute clinical treatment in the trauma center of admission. The radiological results as evaluated by a senior radiologist were extracted from the medical files.

### MRI acquisition and pre-processing

MRI was performed at an average 2.8 years post-injury on a 3 Tesla whole-body unit (Discovery MR750, GE Healthcare, Milwaukee, Wisconsin) using an 8-channel head-coil. Three-dimensional T1-weighted images were acquired using a fast spoiled gradient-echo sequence (176 slices, acquisition matrix 256 × 256, voxel-size 1 × 1 × 1 mm, TR/TE/TI = 8.2/3.2/450 ms, flip angle 15°). Furthermore, two-dimensional echo-planar diffusion-tensor images were acquired in 5 volumes without diffusion weighting and 30 volumes with non-collinear diffusion gradients (b-value = 750 s/mm^2^) in 47 transverse/slightly oblique slices of 2.5 mm thickness (axially angulated parallel to the line connecting the pituitary to the fastigium of the fourth ventricle) covering the whole brain (TR/TE =5000/74 ms). The acquired in-plane resolution was 2.5 × 2.5 mm, reconstructed to 1 × 1 × 2.5 mm. Parallel imaging was applied with an acceleration factor of 2. All processing of MR images was performed using the Functional MRI of the Brain (FMRIB) Software Library (FSL) version 5.0.8 (Jenkinson et al. 2012).

Pre-processing of diffusion-tensor images included correction of artifacts caused by head motion and eddy currents. Diffusion tensor fitting was performed on brain-extracted images to create fractional anisotropy (FA), mean diffusivity (MD), axial diffusivity (AD; largest eigenvalue of diffusion tensor) and radial diffusivity (RD; mean of the two lowest eigenvalues of diffusion tensor) maps, while the quality of the tensor fit was visually inspected on the sum of squared error maps (Behrens et al. 2003). Volumes with motion distortion causing evident artifacts in the SSE maps were removed from the original diffusion-weighted dataset, leading to the removal of 38 volumes (1.7%) in a total of 19 subjects, with a maximum of 6 out of 35 volumes per subject. For these 19 subjects with one or more discarded volumes, pre-processing was repeated on the data without the volumes containing motion artifacts.

#### Volumetric analysis of T1-weighted MRI scans

Brain tissue volume was measured in T1-weighted images, which were visually inspected for scanning artifacts due to head motion. T1 scans of two children in the TC group and two children in the mild^RF+^ TBI group were discarded from volumetric analyses due to severe motion distortion. Volumes of white matter, grey matter and subcortical structures (thalamus, caudate nucleus, putamen, globus pallidum, hippocampus, amygdala and accumbens nuclei in both hemispheres, and the upper brainstem) were estimated using SIENAX (Zhang et al. 2001) and FIRST (Patenaude et al. 2011). Whole brain, grey matter, white matter volumes were normalized for head size and the v-scaling factor resulting from SIENAX was used to normalize subcortical structures as well. Since FIRST produces the volumes of lateralized subcortical structures, left and right volumes of each structure were combined to focus on the diffuse impact of TBI and to reduce the number of group comparisons. Last, putamen and globus pallidum volumes were collapsed to measure the volume of the striatum.

#### Tract-based spatial statistics (TBSS) on DTI data

Voxel-wise statistical analysis of the DTI data was performed using TBSS (Smith et al. 2006). First, the DTI maps (i.e. FA, MD, AD and RD maps) of all subjects were aligned to the most typical subject in the TC group, as determined by non-linear registrations of FA maps between all children in the TC group. Next, the DTI maps were transformed to MNI152 space using non-linear transformation and a white matter skeleton (FA > 0.2) was computed to reduce the confounding effects of misalignment and inter-subject variability in white matter tract anatomy. The DTI data of one child in the moderate/severe TBI group with major brain damage after neural resection during neurosurgery was discarded, because non-linear transformation of the damaged brain led to severe deformation of the brain in MNI space, causing unreliable alignment. The remaining subjects’ DTI maps (*n* = 63) were projected onto the skeleton in standard space and voxel-wise TBBS statistics were performed using randomise on the resulting maps, where threshold-free cluster enhancement and *P*-values as corrected for family-wise error accounted for multiple testing. Last, masks of bilateral white matter tracts were created from the FSL built-in John Hopkins University Atlas and multiplied with the FA skeleton to retrieve the following skeletonized tracts: genu, body and splenium of the corpus callosum (GCC, BCC and SCC, respectively) superior and inferior longitudinal fasciculus (SLF and ILF), inferior frontal occipital fasciculus (IFOF), anterior thalamic radiation (ATR), corticospinal tract (CST), forceps major and minor (FMa and FMi), cingulate and hippocampal parts of the cingulum bundle (CCB and HCB) and uncinate fasciculus (UF).

### Functional outcome

Functional outcome was measured using Common Data Elements for neurocognitive and behavioral outcome in the chronic phase of pediatric TBI (McCauley et al. 2012). General neurocognitive functioning was operationalized as intelligence and measured by a Wechsler Intelligence Scale (WISC)-III (Kort et al. 2005) short-form (involving the Vocabulary and Block Design subtests) estimation of age-standardized full-scale IQ (FSIQ), which has shown high validity and reliability in estimating FSIQ (Sattler 2001). Attention and working memory were measured using the age-standardized score on the Digit Span subtest of the WISC-III (Wechsler 1991). Verbal learning and memory was measured using the Rey Auditory Verbal Learning Test (RAVLT; van den Burg and Kingma 1999). Age-standardized z-scores of total correct responses in the direct recall, delayed recall and recognition conditions were used to measure three aspects of verbal memory: (1) Encoding (i.e. the ability to learn new information), as measured by the direct recall condition score; (2) Retrieval (i.e. the ability to access previously learned material stored in long-term memory), as measured by the difference between the direct recall score and the delayed recall score; and (3) Consolidation (i.e. the ability to store information in long-term memory), as measured by the difference between the the delayed recall score and the recognition score.

Behavioral functioning was measured using parent and teacher ratings of internalizing problems (e.g. symptoms of anxiety and depression) and externalizing problems (e.g. aggression and symptoms of conduct disorder), obtained using the Child Behavior Checklist and the Teacher Rating Form (Verhulst and van der Ende 2013). Age- and gender- standardized T-scores of parents and teacher ratings were averaged to yield composite scores of internalizing and externalizing problems. For clarity reasons, all functional outcome scores were transformed to z-scores where lower values intuitively correspond to poorer neurocognitive performance/less behavior problems.

### Procedure

The current study represents a follow-up of an earlier investigated sample of children (for more information on the recruitment of this sample, see Königs et al. 2015a). All 123 children that were eligible for the current follow-up (TBI: *n =* 67; TC: *n =* 56), were sent an information letter and were contacted by telephone to provide additional information about the study. Eleven children were not reached (TBI: *n =* 8; TC: *n =* 3) and 36 declined participation (TBI: *n =* 17; TC: *n =* 19). Main reasons not to participate were: not interested (TBI: 41%; TC: 32%), objection to MRI (TBI: 0%; TC: 21%) and no time (TBI: 24%; TC: 21%). A total of 12 children were excluded from participation due to dental braces that were incompatible with MRI (TBI: *n =* 2; TC: *n =* 6), claustrophobia (TBI: *n =* 2; TC: *n =* 1) or no show (TBI: *n =* 1; TC: *n =* 0). The remaining children in the TBI and TC groups (TBI: *n =* 37; TC: *n =* 27) did not differ from their respective recruitment cohorts in terms of age, gender and SES (TBI: *p*s ≥ .28; TC: Ps ≥ .07), or GCS score (TBI: *p* = .68).

Written informed consent was provided by parents and children aged >11 years. Trained examiners administered the neurocognitive tests in a fixed order, while parents filled out questionnaires in a waiting room. Subsequently, children were made familiar with the MRI procedure using a simulation scanner before actual MRI scanning was performed in the VU University Medical Center. Neurocognitive testing and MRI scanning were performed on the same day for all participants. Time since injury in the TBI sample ranged between 0.8 and 6.2 years. All procedures performed in this study were in accordance with the ethical standards of medical ethical committee of the VU University Medical Centre (NL37226.029.11) and with the 1964 Helsinki declaration and its later amendments or comparable ethical standards.

### Statistical analysis

Statistical analyses were performed using SPSS 22.0 (IBM Corp. 2011). The dependent variables were screened for outliers (−3.29 > z-score > 3.29), which were rescaled using Windsorizing (Tabachnick and Fidell 2012). The TC, mild^RF+^ TBI and moderate/severe TBI groups were compared on demographic variables, injury-related variables and the prevalence of diagnosed psychiatric or learning disorders using ANOVA and chi-square tests, as appropriate.

First, we investigated the effects of TBI severity on the assessed neuroimaging parameters derived from acute CT-scans, and post-acute T1-weighted MRI and DTI. The prevalence of acute CT-scans with evident intracranial pathology was compared between groups using chi-square tests. The effects of TBI severity (TC, mild^RF+^ TBI and moderate/severe TBI) on the volumes of white matter, grey matter and subcortical structures in T1-weighted MRI scans were assessed using ANOVA. Next, we investigated the effect of TBI severity on whole brain white matter integrity (as assessed by DTI scans) using ANOVA on mean whole skeleton FA. In addition, regional effects of TBI on white matter integrity were assessed by voxel-wise comparisons of the skeletonized FA maps between all groups using TBSS. If one or more cluster(s) with regional group-differences in FA was/were observed, we further extracted the mean FA, MD, AD and RD values from each cluster, and used ANOVA on these DTI parameters to track down the origin of the observed effect of TBI severity on FA. A pattern of increased MD, decreased AD and/or increased RD will be interpreted as indicative of axonal degeneration and/or demyelination (Budde et al. 2011). If effects of TBI severity on MD, AD and/or RD were observed, the spatial distribution of this effect was investigated using TBSS on the relevant skeletonized maps. Overlap between each white matter tract (GCC, BCC, SCC, SLF, ILF, IFOF, ATR, CST, FMa, FMi, CCB, HCB and UF) and (the) cluster(s) of affected white matter tracts was used to assess the contribution of each white matter tract to the neuropathology of TBI (i.e. overlap as a percentage of the total cluster size of affected white matter tracts in voxels) and the extent to which each tract was affected by the neuropathology of TBI (i.e. overlap as a percentage of the total skeletonized white matter tract size).

Second, the impact of TBI severity on aspects of functional outcome was assessed. ANOVAs assessed the main effect of TBI severity on FSIQ as a measure of intelligence, Digit Span score as a measure of attention and working memory, RAVLT Encoding, Retrieval and Consolidation scores as aspects of verbal learning and memory, and ratings of internalizing and externalizing behavior problems as measures of behavioral functioning. All analyses with main effects of TBI severity were subjected to subsequent pairwise comparisons between groups (TC, mild^RF+^ TBI and moderate/severe TBI) using post-hoc LSD testing.

Third, the predictive value of neuroimaging parameters was investigated. Using Pearson correlations in the whole sample, relations were assessed: (1) among neuroimaging parameters with observed effects of TBI; and (2) between these neuroimaging parameters on the one hand, and neurocognitive and behavioral variables with observed effects of TBI on the other hand. Regional associations between FA within the cluster(s) of affected white matter tracts and aspects of functional outcome (with observed effects of TBI) were assessed using voxel-wise linear regression in TBSS, where threshold-free cluster enhancement and *p* values as corrected for family-wise error accounted for multiple testing. Scatter plots were produced for all significant associations between neuroimaging parameters and functional outcome. Finally, the role of continuous neuroimaging parameters in the observed effects of TBI on functional outcome were assessed using mediation models (Hayes 2013). All statistical analyses were two-sided, α was set at .05 and effect sizes of group differences were expressed as Cohen’s *d*.

## Results

### Patient characteristics

Demographics, injury-severity variables and prevalence of psychiatric and learning disorders are displayed for all groups in Table 1. There were no group differences on demographic variables (*p*s ≥ .17), except for lower SES in the mild^RF+^ TBI and moderate/severe TBI groups as compared to the TC group (*p*s ≤ .034). Regarding injury-related variables, the mechanism of injury was more likely to be a fall (as opposed to a traffic accident) in the TC group as compared to both the mild^RF+^ TBI and moderate/severe TBI groups (Ps < .02). As expected, the moderate/severe TBI group had lower GCS scores than the mild^RF+^ TBI group (*p* < .001), while higher prevalence of neurosurgery and longer hospital stay were observed as compared to both the mild^RF+^ TBI group and TC group (*p* ≤ .009). The TC group had higher prevalence of extracranial fractures and orthopedic surgery than both the mild^RF+^ TBI group (*ps* < .001) and the moderate/severe TBI group (*p*s < .001). Last, there were no group differences in the observed prevalence of psychiatric disorders (i.e. attention-deficit/hyperactivity disorder) or learning disorders (i.e. dyslexia; *p*s > .05).

**Table 1:** Demographic and injury-related data in the TC, mild^RF+^ TBI and moderate/severe TBI groups

*n*	Groups			Contrasts
	TC	Mild RF+ TBI	Moderate/Severe TBI	
	27	20	17	
Demographics				
Males, n (%)	12 (44)	13 (65)	10 (59)	NS
Age at testing in y	10.2 (1.5)	10.5 (1.8)	10.0 (1.4)	NS
SES	6.3 (1.1)	5.5 (1.3)	5.0 (1.1)	TC > M, MS
Injury-related information				
Age at injury in y	7.5 (2.2)	7.7 (2.3)	7.0 (1.9)	NS
Injury mechanism				
Fall, n (%)	23 (85)	11 (55)	8 (47)	TC > M, MS
Traffic accident, n (%)	4 (15)	9 (45)	9 (53)	TC < M, MS
Lowest GCS		14.5 (0.7)	8.4 (2.8)	M > MS
Hospital Stay in *d*	2.5 (2.0)	3.5 (2.4)	9.6 (9.3)	TC, M < MS
Time since injury in y	2.7 (1.0)	2.8 (1.1)	3.0 (1.4)	NS
Range	*1.0–4.5*	*0.8–5.3*	*1.0–6.2*	
Extracranial fracture, n (%)	23 (85)	4 (20)	2 (12)	TC > M, MS
> 1 Extracranial fractures, n (%)	3 (11)	1 (5)	0 (0)	NS
Orthopedic surgery, n (%)	22 (82)	2 (10)	0 (0)	TC > M, MS
Neurosurgery, n (%)	0 (0)	0 (0)	5 (29)	TC, M < MS
Diagnosed conditions				
Psychiatric disorder, n (%)	1 (4)	2 (10)	1 (6)	NS
Learning disorder, n (%)	2 (7)	4 (20)	0 (0)	NS

Data reflect mean (SD), unless otherwise indicated. *TC* traumatic control; *TBI* traumatic brain injury; *RF* risk factor; *SES* socio-economic status; *NS* not significant; *y* years; *d* days; *GCS* Glasgow Coma Scale; *M* mild^RF+^ TBI group; *MS* moderate/severe TBI group

### Acute neuroimaging using CT

Group comparisons on the results of acute CT-scans are provided in Table 2. As expected, the mild^RF+^ TBI group and the moderate/severe TBI group had higher prevalence of intracranial pathology than the TC group (*p* < .001). Furthermore, children with moderate/severe TBI had higher prevalence of intracranial pathology than the mild^RF+^ TBI group (*p* = .035).

**Table 2:** Neuroimaging data in the TC, mild^RF+^ TBI, moderate/severe TBI groups

n*	Groups			ANOVA		
	TC	Mild RF^+^ TBI	Moderate/Severe TBI	F(2,62)	*p*	Contrasts
	27	20	17			
Acute CT-scan						
Intracranial Pathology, n (%)	0 (0)	6 (35)	11 (65)			TC < M < MS
Normalized Brain Volume (cm^3^)						
White matter	701.0 (28.1)	680.4 (28.1)	675.6 (32.4)	4.5	**.016**	TC > M, MS
Grey matter	1070.0 (38.0)	1044.5 (50.6)	1051.8 (53.3)	1.8	.18	
Normalized Subcortical Volume (cm^3^)						
Thalamus	21.8 (1.2)	21.2 (1.4)	21.0 (2.1)	1.5	.23	
Caudate Nuclei	10.1 (1.3)	10.7 (0.9)	9.8 (1.7)	3.2	.050	
Striatum	19.5 (1.7)	18.5 (1.5)	18.9 (2.0)	1.7	.19	
Hippocampus	10.4 (9.3)	10.0 (1.1)	10.6 (1.3)	0.2	.82	
Amygdala	3.2 (0.5)	3.1 (0.5)	3.3 (0.7)	0.4	.64	
Accumbens Nuclei	1.4 (0.3)	1.4 (0.2)	1.3 (0.4)	1.1	.33	
Upper Brainstem	26.1 (1.9)	25.6 (2.4)	26.2 (3.2)	0.3	.72	
DTI Parameters						
FA whole brain skeleton	.421 (.015)	.420 (.020)	.407 (.019)	4.1	**.022**	TC, M > MS
FA whole cluster	.468 (.018)	.458 (.023)	.432 (.021)	15.4	**<.001**	TC, M > MS
MD whole cluster (10^−5^ mm^2^/s)	84.7 (2.4)	85.3 (2.8)	86.8 (2.7)	3.2	**.049**	TC < MS
AD whole cluster (10^−5^ mm^2^/s)	133.0 (2.2)	132.6 (2.4)	131.6 (2.8)	1.8	**.**18	NS
RD whole cluster (10^5^ mm^2^/s)	60.6 (2.8)	61.6 (3.5)	64.4 (3.2)	7.5	**.001**	TC, M < TC

Data reflect mean (SD), unless otherwise indicated. Bold values pertain to significant results. *TC* traumatic control; *TBI* traumatic brain injury; *RF* risk factor; *NS* not significant; *FA* fractional anisotropy; *MD* mean diffusivity; *AD* axial diffusivity; *RD* radial diffusivity; *M* mild^RF+^ TBI group; *MS* moderate/severe TBI group

*Missing values for brain volumes (*n* = 4) and DTI parameters (*n* = 1)

### Volumetric analysis of T1-weighted MRI scans

The volumetric analyses of white matter, grey matter and subcortical structures are displayed in Table 2. The results showed a significant main effect of TBI severity on white matter volume, but not on grey matter volume. Subsequent pairwise group comparisons revealed that the mild^RF+^ TBI group and moderate/severe TBI group both had smaller white matter volume than the TC group (*p* = .029, *d* = −0.74 and *p* = .009, *d* = −0.80, respectively). No significant main effects of TBI severity on the volumes of subcortical structures (i.e. thalamus, caudate nuclei, striatum, hippocampus, amygdala, nucleus accumbens and brainstem) were found (*p*s ≥ .05). Together, these findings indicate that the effects of mild^RF+^ TBI and moderate/severe TBI primarily manifest on the volume of white matter, rather than grey matter or subcortical structures.

### White matter integrity in DTI

**Fig. 1:**
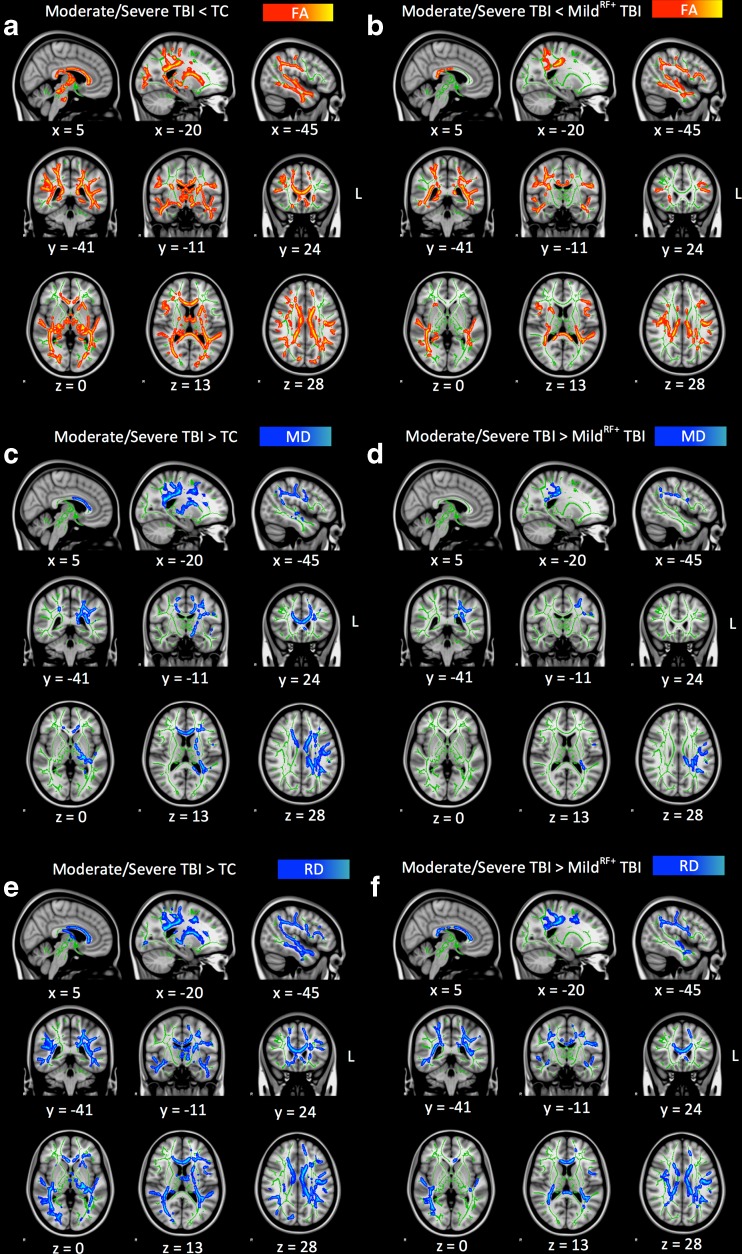
Voxel-wise comparison of FA, MD and RD maps using threshold-free cluster enhanced correction in TBSS. *Note.* Results of voxel-wise group comparisons showing the parts of the whole brain skeleton (at FA > 0.2, in green) with differences in terms of FA, MD and RD between the moderate/severe TBI group as compared to the TC group (Panels **a**, **c** and **e**, respectively) and mild^RF+^ TBI group (Panels **b**, **d** and **e**, respectively). Lower values in the moderate/severe TBI group as compared to other groups are displayed in *red-yellow*, while higher values are displayed in *blue*-*lightblue*. The results are overlaid on a MNI152 1 mm T1 brain in radiological convention (*right* = *left*), and for visualization purposes, regions in the whole brain skeleton with significant group differences were ‘thickened’ towards the full width of the white matter tract. *TC* trauma control group; *TBI* traumatic brain injury; *FA* fractional anisotropy; *MD* mean diffusivity; *RD* radial diffusivity

A significant effect of TBI severity on mean FA in the whole brain skeleton was found (Table 2). Subsequent pairwise group comparisons revealed that the moderate/severe TBI group had lower FA in the whole brain skeleton than both the TC group (*p* < .001, *d* = −1.88) and the mild^RF+^ TBI group (*p* < .001, *d* = −1.20), while no significant difference between the mild^RF+^ TBI group and TC group was observed (*p* = .10, *d* = −0.50). These findings indicate that children with moderate/severe TBI have diffuse white matter abnormalities. To localize regional effects of TBI on FA, voxel-wise group comparisons on the skeletonized FA maps were performed using TBSS. This analysis revealed one large cluster of lower FA in the moderate/severe TBI group as compared to the TC group as well as the mild^RF+^ group (Fig. 1a, b).

Analyses aimed at tracking down the origin of the effect of moderate/severe TBI on FA in the cluster of affected white matter tracts (Table 2), showed that the moderate/severe TBI group had higher MD and RD than the TC group (*p* = .015, *d* = 0.84 and *p* < .001, *d* = 1.32, respectively) and higher RD as compared to the mild^RF+^ TBI group (*p* < .001, *d* = 0.85). No differences in AD were observed between the moderate/severe TBI group and the TC group (*p* = .07, *d* = −0.60) or mild^RF+^ group (*p* = .20, *d* = −0.43).

Last, we investigated the regional effects of moderate/severe TBI on the skeletonized MD and RD maps as compared to both the TC group and the mild^RF+^ TBI group. Large clusters of higher MD and RD were observed in the moderate/severe TBI group as compared to the TC group as well as the mild^RF+^ TBI group (Fig. 1c-f), with high correspondence to the identified cluster of decreased FA (Fig. 1a, b).

Analyses aimed at identifying the white matter tracts involved in the neuropathology of moderate/severe TBI, revealed that all of the assessed bilateral white matter tracts were affected (see Table 3). More specifically, overlap between each white matter tract and the cluster of affected white matter tracts was used to assess the contribution of each white matter tract to the neuropathology of moderate/severe TBI (i.e. overlap as a percentage of the total cluster size of affected white matter tracts) and the extent to which each tract was affected by the neuropathology of moderate/severe TBI (i.e. overlap as a percentage of the total white matter tract size). Parts of the SLF, ILF and IFOF most prominently contributed to the neuropathology of moderate/severe TBI, while the BCC, SCC and HCB were least prominently involved. Otherwise, the GCC, BCC and SCC were most extensively affected by moderate/severe TBI, while the UF, FMi, and ATR were least extensively affected. Together, these findings indicate that children with moderate/severe TBI have widespread white matter abnormalities characterized by decreased FA, increased MD and increased RD. Furthermore, the results indicate that the SLF, ILF and IFOF are primarily involved in the white matter pathology of moderate/severe pediatric TBI, while the GCC, BCC and SCC most are the most extensively affected tracts.

**Table 3:** White matter tract involvement in the neuropathology of moderate/severe TBI as measured with DTI

White Matter Tract	% of Cluster	% of Tract Affected
Superior Longitudinal Fasciculus (SLF)	24.7	33.7
Inferior Longitudinal Fasciculus (ILF)	23.7	44.7
Inferior Frontal Occipital Fasciculus (IFOF)	20.3	30.7
Anterior Thalamic Radiation (ATR)	15.1	25.1
Cortical Spinal Tract (CST)	8.0	30.9
Forceps Major (FMa)	7.8	39.6
Genu of Corpus Callosum (GCC)	5.4	68.8
Forceps Minor (FMi)	4.8	23.1
Cingulate part of Cingulum Bundle (CCB)	3.7	32.7
Uncinate Fasciculus (UF)	3.7	14.5
Body of Corpus Callosum BCC	2.9	66.7
Splenium of Corpus Callosum (SCC)	2.7	51.8
Hippocampal part of Cingulum Bundle (HCB)	2.5	32.1

### Functional outcome

Analysis of neurocognitive functioning (Table 4) revealed significant main effects of TBI severity on FSIQ, Digit Span and RAVLT Encoding scores, but not on RAVLT Retrieval and Consolidation scores. These findings indicate that TBI severity affects intelligence, attention and working memory and the encoding of information into verbal memory. Subsequent pairwise group comparisons indicated that the mild^RF+^ TBI and moderate/severe TBI group had lower FSIQ (*p* = .003, *d* = −0.93 and *p* = .008, *d* = −0.92), Digit Span (*p* = .006, *d* = −0.92 and *p* = .017, *d* = −0.77) and RAVLT Encoding scores (*p* = .040 , *d* = −0.63 and *p* = .009, *d* = −0.89) than the TC group, while no significant differences between the mild^RF+^ TBI and moderate/severe TBI groups were observed (*ps* ≥ .53, *d*s ≥ −0.21).

**Table 4:** Functional outcome in the TC, mild^RF+^ TBI and moderate/severe TBI groups

*n*	Groups			ANOVA		
	TC	Mild RF^+^ TBI	Moderate/Severe TBI	F(2,62)	*p*	Contrasts
	27	20	17			
Neurocognitive functioning						
FSIQ	0.47 (0.82)	-0.37 (1.04)	-0.32 (0.95)	6.0	**.004**	TC > M, MS
Digit Span	0.44 (0.87)	-0.36 (0.91)	-0.28 (1.08)	5.1	**.009**	TC > M, MS
RAVLT Encoding	0.40 (0.90)	-0.20 (1.05)	-0.40 (0.92)	4.2	**.019**	TC > M, MS
RAVLT Retrieval	-0.03 (0.90)	0.03 (0.90)	0.02 (0.85)	0.0	.97	
RAVLT Consolidation	0.05 (0.92)	-0.04 (1.15)	-0.03 (0.98)	0.1	.94	
Behavior Problems						
Internalizing Problems	-0.37 (0.88)	0.20 (0.77)	0.46 (1.29)	5.5	**.006**	TC < M, MS
Externalizing Problems	-0.37 (0.88)	0.58 (0.84)	-0.10 (1.10)	6.2	**.003**	TC, MS < M

Z scores are reported. Data reflect mean (SD) unless otherwise indicated. *TC* traumatic control; *TBI* traumatic brain injury; *RF* risk factor; *FSIQ* full-scale IQ; *M* mild^RF+^ TBI group; *MS* moderate/severe TBI group; *RAVLT* Rey Auditory Verbal Learning Test

With regard to behavioral functioning, significant effects of TBI severity on ratings of internalizing problems and externalizing problems were observed (Table 4). Subsequent pairwise group comparisons revealed that, as compared to the TC group, the mild^RF+^ TBI and moderate/severe TBI groups had higher ratings of internalizing problems (*p* = .023, *d* = 0.85 and *p* = .003, *d* = 0.92). With regard to externalizing problems, the mild^RF+^ TBI group had higher ratings as compared to the TC group (*p* = .001, *d* = 1.13), while the moderate/severe TBI group had not (*p* = .34, *d* = 0.29). Comparisons between the mild^RF+^ TBI group and moderate/severe TBI group revealed no significant differences on ratings of internalizing problems (*p* = .41, *d* = 0.25), while the mild^RF+^ TBI group had higher ratings of externalizing problems as compared to the moderate/severe TBI group (*p* = .03, *d* = 0.73).

### Neuroimaging parameters and functional outcome

Subsequent analyses investigated the correspondence among neuroimaging parameters with observed effects of TBI severity (i.e. intracranial pathology on acute CT-scans, white matter volume on T1-weighted MRI and FA in the cluster of affected white matter tracts). Intracranial pathology on acute CT was associated with smaller white matter volume (*r* = −0.29, *p* = .03) as well as lower FA in the cluster of white matter tracts affected by moderate/severe TBI (*r* = −0.26, *p* = .05). In turn, smaller white matter volume was associated with lower FA in the cluster of affected white matter tracts (*r* = .39, *p* = .002). These results indicate that the assessed neuroimaging parameters have a small to moderate degree of correspondence, and confirm that CT, volumetric analysis of T1-weighted MRI scans and DTI measure only partially overlapping aspects of the TBI neuropathology.

**Fig. 2:**
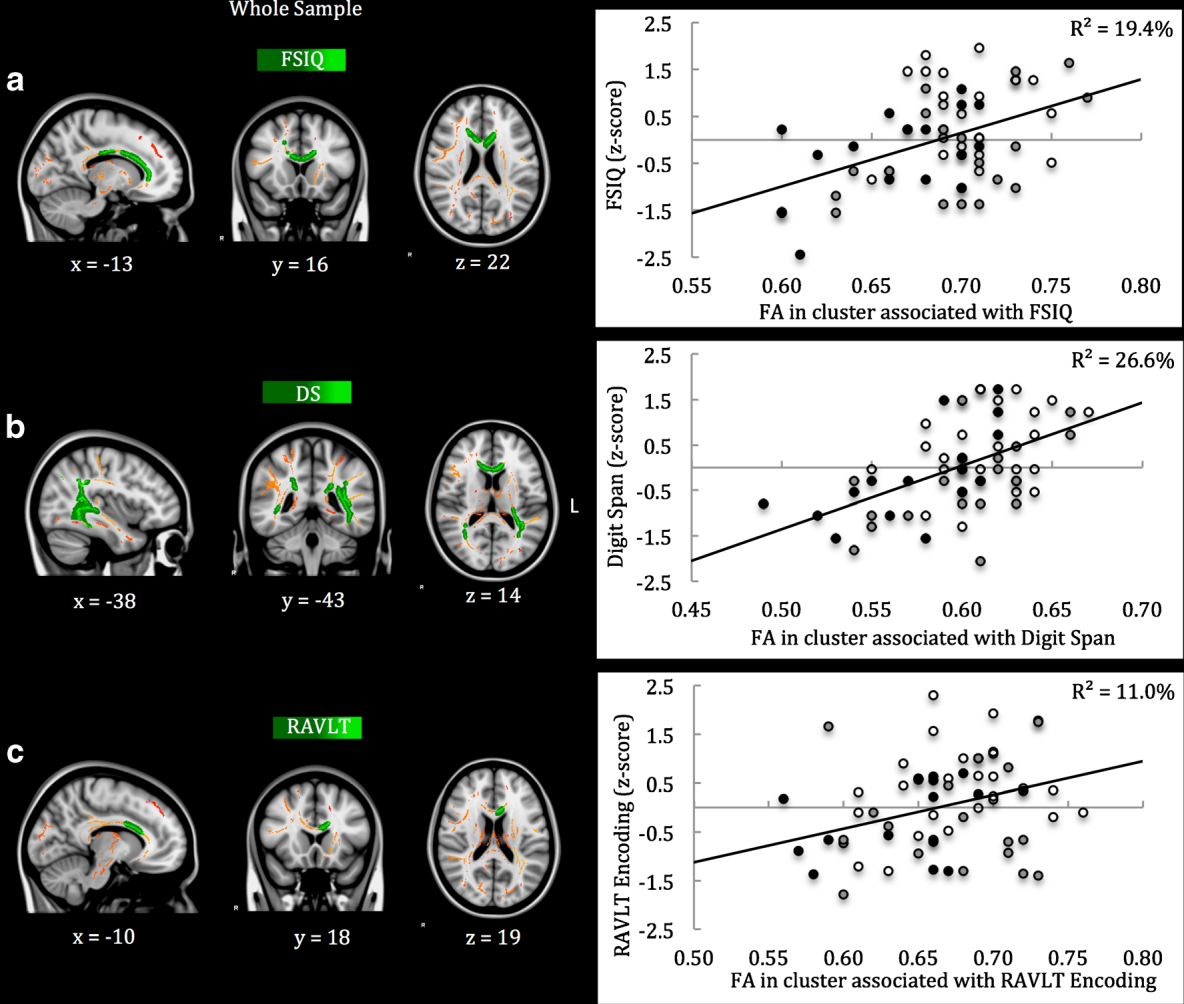
Regional associations between FA and functional outcome using threshold-free cluster enhancement correction in TBSS. *Note.* Regional associations are displayed (in *green*-*light green*) between FA in the cluster of affected white matter tracts and FSIQ (Panel **a**), Digit Span score (Panel **b**) and RAVLT Encoding score (Panel **c**). The cluster of white matter tracts affected by moderate/severe TBI is displayed in *red*-*yellow*. The results are overlaid on a MNI152 1 mm T1 brain in radiological convention (*right* = *left*), and for visualization purposes, regions in the cluster of affected white matter tracts with significant associations to functional outcome were ‘thickened’ towards the full width of the white matter tract. Color of the data point in the scatter plots refers to the trauma control group (*white*), mild^RF+^ TBI group (*grey*) and moderate/severe TBI group (*black*). *FSIQ* full-scale IQ; *DS* Digit Span; *RAVLT* Rey Auditory Verbal Learning Test

Furthermore, the relations between these neuroimaging parameters and aspects of functional outcome with observed effects of TBI severity were investigated (i.e. FSIQ, Digit Span and RAVLT Encoding scores, and ratings of internalizing and externalizing problems). This analysis revealed that intracranial pathology on acute CT scans was only related to higher ratings of internalizing problems (*r* = .35, *p* = .004). White matter volume showed no significant relations to aspects of functional outcome at all (*r*s ≤ .23, *p*s ≥ .07). In contrast, lower FA in the cluster of affected white matter tracts was associated with lower FSIQ (*r* = .29, *p* = .02), lower Digit Span score (*r* = .41, *p* < .001) and higher ratings of internalizing problems (*r* = −.26, *p* = .04; also see eFigure 1). Regional associations between FA within the cluster of affected white matter tracts and aspects of functional outcome with observed effects of TBI were investigated using TBSS (Fig. 2). The results show that lower FA in parts of: (A) the GCC, BCC and CCB was associated with lower FSIQ (*r* = .45, *p* < .001); (B) the GCC, BCC, SCC, ILF, IFOF and SLF was associated with lower Digit Span scores (*r* = .53, *p* < .001); and (C) the GCC and BCC was associated with lower RAVLT Encoding scores (*r* = .35, *p* = .005); while no regional associations between FA and ratings of internalizing or externalizing problems were identified.

Last, mediation models (see eFigure 2) were used to investigate the potentially mediating role of FA in the relation between moderate/severe TBI and functional outcome (FSIQ, Digit Span and RAVLT Encoding scores). As expected, these analyses showed that moderate/severe TBI was related to lower FA in the clusters with regional associations to functional outcome (Path A, see Fig. 2 for clusters) and poorer FSIQ, Digit Span and RAVLT Encoding scores (Path C). Likewise, lower FA in these clusters was related with lower FSIQ, Digit Span and RAVLT Encoding scores (Path B). Notably, lower FA in the clusters associated with FSIQ, Digit Span and RAVLT Encoding scores (Fig. 2), fully mediated the effects of moderate/severe TBI on FSIQ (B [SE] = 0.10 [0.06], 95% confidence interval [CI] = −0.01 to −0.27), Digit Span scores (B [SE] = 0.17 [0.06], 95%-CI = 0.08 to 0.31) and RAVLT Encoding scores (B [SE] = 0.12 [0.06], 95%-CI = 0.03 to 0.27), respectively (Path C′). Taken together, the results indicate that although acute and post-acute neuroimaging parameters are interrelated, DTI shows superior predictive value for functional outcome. More specifically, the results suggest that the diffuse impact of moderate/severe TBI on white matter integrity is, at a regional level, associated with intelligence, working memory and encoding of information in verbal memory, where disrupted integrity in parts of the CC was consistently associated with poorer neurocognitive functioning.

### Analysis of possible confounders

Lower SES in the mild^RF+^ TBI group and moderate/severe TBI group as compared to the TC group could have confounded some of the reported group differences, since SES was also related to intracranial pathology on CT scans, FA in the cluster of affected white matter tracts, FSIQ, Digit Span score and RAVLT Encoding scores (*r*s ≥ 25, *p*s < .05). Therefore, the analyses were repeated after matching children with TBI to the TC group on SES. In this matching procedure, a child with (mild^RF+^ or moderate/severe) TBI was matched to each child in the TC group on SES (within a bandwidth of 1 unit on the 8-point SES scale), while counterbalancing for TBI severity in the matched TBI group. If multiple matches were possible using these criteria, we also matched for age and gender. The resulting matched TBI group and the TC group showed no significant differences on age, gender and SES (*p*s ≥ .10) and the matched TBI group had a balanced representation of mild^RF+^ TBI (52%) and moderate/severe TBI (48%). Repeating the analyses using the matched groups replicated all reported group differences (data available with first author), with the single exception of ratings of externalizing problems (*p* = .10, *d* = 0.46). Together, these findings indicate that SES did not account for the reported group differences, with the possible exception of higher ratings of externalizing problems in the mild^RF+^ TBI group as compared to the TC group.

## Discussion

This study investigated the neuropathology of mild^RF+^ to moderate/severe TBI in children and the predictive value of neuroimaging parameters (i.e. acute CT-scans and post-acute MRI techniques) for functional outcome. The results indicate that mild^RF+^ to moderate/severe TBI causes white matter volume loss that is indicative of chronic white matter atrophy, while Tract-Based Spatial Statistics on DTI data revealed that moderate/severe TBI is additionally associated with widespread disruption of white matter integrity (i.e. reduced FA). Among the assessed neuroimaging parameters, DTI measures of white matter integrity (i.e. FA) most consistently displayed predictive value for functional outcome in children with TBI, both in terms of neurocognitive and behavioral functioning. The results of this study underline the potential clinical relevance of DTI parameters for the prognosis of functional outcome after pediatric TBI.

Analysis aimed at the neuropathology of TBI, indicated that 35% of children with mild^RF+^ TBI had intracranial pathology on acute CT-scans. Volumetric analysis of post-acute T1-weighted MRI scans showed no effects of mild^RF+^ TBI on cortical grey matter or subcortical structures, whereas children with mild^RF+^ TBI did show decreased white matter volume as compared to children with TC injury. To our best knowledge, this is the first study to show that mild^RF+^ TBI causes persisting white matter abnormality. Nevertheless, this finding is in line with our previous research showing that children with mild^RF+^ TBI are at risk of a range of neurocognitive impairments (i.e. general neurocognitive functioning, attention and visual integration) and behavior problems (i.e. internalizing and externalizing problems; Königs et al. 2015a, b). Together, these findings call for careful clinical screening for adverse neurocognitive and behavioral outcome in children with mild^RF+^ TBI.

It was surprising to observe an impact of mild^RF+^ TBI on white matter volume in the absence of corresponding effects on white matter integrity from DTI analyses. A comparable dissociation between white matter volume and white matter integrity has previously been reported in a longitudinal study investigating CC development between 3 to 12 months after complicated mild to severe pediatric TBI (Wu et al. 2010). The findings from that study suggested abnormal post-injury development of white matter at the macrostructural level (i.e. CC volume loss) thought to be caused by Wallerian degeneration as a net response to total diffuse damage to axons as well as neurons, in combination with microstructural white matter changes (i.e. FA increase) thought to reflect continued development of myelin and possible compensatory regenerative processes. In line with this reasoning, we speculate that the observed effect of mild^RF+^ TBI on white matter volume may have arisen as a net (Wallerian) result of subthreshold axonal as well as cortical injury. Alternatively, the absence of effects of mild^RF+^ TBI on white matter integrity (i.e. DTI parameters) in the post-acute phase may reflect successful regeneration of white matter at the microstructural level.

As expected, moderate/severe TBI was associated with a high prevalence of intracranial pathology on acute CT-scans (65%). Furthermore, children with moderate/severe TBI had decreased white matter volume as compared to children with TC injury, while no effects on the volumes of grey matter and subcortical structures were observed. The results of DTI analyses further indicated that children with moderate/severe TBI in addition have widespread disruptions of white matter integrity (i.e. decreased FA, and increased MD and RD), likely reflecting axonal degeneration and/or reduced myelin density (Budde et al. 2011). We further found that all assessed white matter tracts were affected by moderate/severe TBI, where parts of the SLF, ILF and IFOF were most prominently involved in the white matter pathology of moderate/severe pediatric TBI (constituting between 20 and 25% of affected white matter), while the most widespread effects of moderate/severe TBI manifested in the GCC, BCC and SCC (between 52 and 69% of tract affected). Together, these findings indicate that moderate/severe TBI in children is associated with a high risk of intracranial pathology, has a persisting detrimental impact on white matter volume and causes widespread disruption of white matter integrity. These findings are in line with the existing literature on the neuropathology of moderate to severe TBI (Sharp et al. 2014). Regarding the time interval between injury and MRI scanning in the children with TBI (0.8–6.2 years), the observed effects of TBI on white matter volume and DTI parameters may reflect not only the direct impact of TBI on white matter, but also the influence of TBI on post-injury white matter development (Ewing-Cobbs et al. 2016).

Neuroimaging parameters with observed sensitivity for TBI (i.e. acute CT, post-acute T1-weighted MRI and DTI) had small to moderate correspondence, emphasizing that these measures clearly tap into different aspects of the TBI neuropathology. With regard to the predictive value of neuroimaging parameters for functional outcome, evidence for intracranial pathology on acute CT scans was only related to higher ratings of internalizing problems, while white matter volume was not related to any aspect of functional outcome. In contrast, lower FA in the cluster of affected white matter was related to poorer neurocognitive functioning (i.e. intelligence, attention and working memory) and behavioral functioning (i.e. internalizing problems). Voxel-wise regression furthermore identified regional associations between lower FA and poorer intelligence (i.e. GCC, BCC and CCB), attention and working memory (i.e. GCC, BCC, SCC, SLF, ILF and IFOF) and encoding in verbal memory (i.e. GCC and BCC). These clusters of lower FA were subsequently found to -in statistical terms- fully mediate the effects of moderate/severe TBI on intelligence, attention and working memory, and encoding in verbal memory, respectively. Taken together, these results confirm the idea that the neuropathology of moderate/severe TBI is primarily characterized by the disruption of white matter integrity (Sharp et al. 2014). The current results further extend the literature from adult and adolescent TBI (Adamson et al. 2013; Haberg et al. 2015; Kinnunen et al. 2011), by showing that specific white matter tracts, consistently involving aspects of the corpus callosum, are likely to underlie a range of neurocognitive impairments after moderate/severe TBI. Last, the findings from this study also indicate that DTI has superior consistency in terms of the predictive value for functional outcome after pediatric TBI, as compared to more conventional neuroimaging parameters (i.e. acute CT scanning and volumetrics of T1-weighted MRI scans).

This study had some weaknesses. First, clinical research in the hospital setting is generally limited by a lack of pre-injury measurements of functioning, and therefore we relied on (suboptimal) group-wise comparisons in a cross-sectional design to assess the average impact of (mild^RF+^ and moderate/severe) TBI on brain structure and functional outcome. Furthermore, we assessed the predictive value of post-acute neuroimaging for functional outcome in a partly cross-sectional design, while the study of the prognostic value of neuroimaging parameters calls for a prospective longitudinal design after TBI. Given the contrasting effects of TBI on DTI parameters in the acute vs. post-acute phase (Roberts et al. 2016), the influence of time between injury and scanning on the prognostic value of DTI parameters is an important issue that remains to be investigated in future studies. Nevertheless, the results from this study highlight the sensitivity of DTI for functional outcome, relative to acute CT-scans and volumetric analysis of post-acute T1-weighted MRI scans. Second, the mild^RF+^ TBI group, moderate/severe TBI group and TC group consisted of relatively small samples, which may have limited statistical power for more subtle effects. For example, the difference between the mild^RF+^ TBI group and TC group on FA in the cluster of affected white matter tracts did not reach conventional levels of significance, despite a considerable effect size indicating lower FA in the mild^RF+^ TBI group (Cohen’s *d* = −0.50). Third, this study focused on the diffuse effects of TBI on the brain, while pediatric TBI also involves focal brain pathology with distinct heterogeneity in the severity and location of lesions (Bigler et al. 2013a). The whole brain and (group-based) ROI analyses that were used in this study are unlikely to systematically capture the relation between *focal* TBI pathology and functional outcome on the brain. However, the heterogeneity of focal pathology may also reduce the predictability of their consequences for functional outcome, while the current study shows that the diffuse impact of TBI on white matter integrity is consistently associated with functional outcome. Fourth and last, the tools used for the analysis of brain volume and white matter integrity have some specific limitations. With regard to volumetric analysis, the scan-rescan reliability of volume estimation has been shown to differ between neural structures (Morey et al. 2010). Some structures typically have low reliability (e.g. amygdala, accumbens) that in turn may have increased the error of measurement in the assessment of these brain volumes. With regard to TBSS, skeletonizing white matter tracts is an essential step in reducing the influence of misalignment across subjects, but also introduces anatomical inaccuracy in the FA skeleton and disregards the impact of neuropathology on the perimeter of white matter tracts (for an in-depth discussion of methodological considerations on TBSS, see Bach et al. 2014). Strong points of this study include: (1) the use of a TC group to control for pre-injury risk factors of trauma and psychological effects of hospitalization and medical procedures (Max et al. 1998); (2) the assessment of a wide range of acute and post-acute neuroimaging techniques; (3) the use of TBSS to allow a data-driven, model-free investigation of regional effects of TBI on white matter integrity, instead of a theory-driven approach (i.e. ROI analysis); (4) the investigation of TBI neuropathology in relation to crucial aspects of functional outcome in children, as assessed using Common Data Elements for outcome of pediatric TBI to maximize the clinical and research utility of the current findings; and (5) the use of a confounding analysis (which showed that group differences in SES were unlikely to account for the main findings), whilst the main findings were based on the (unmatched) samples with best representative value for the pediatric TBI population.

In conclusion, this study confirms previous literature suggesting that the core neuropathology of pediatric TBI manifests in the brain’s white matter. The results extend the literature by revealing evidence indicating that children with mild^RF+^ TBI are at risk of persisting white matter atrophy. Furthermore, we showed that white matter integrity as measured by DTI has superior predictive value for functional outcome, relative to acute CT-scanning and volumetric analysis on post-acute conventional (T1-weighted) MRI. More specifically, the results of this study suggest that regional disruption of white matter integrity in the corpus callosum (GCC and BCC) may importantly underlie neurocognitive impairments in children with moderate/severe TBI. Together, these findings emphasize the potential clinical relevance of DTI as an important prognostic factor of functional outcome of TBI in children, especially in terms of neurocognitive functioning.

## Electronic supplementary material

eFigure 1 Scatter plots of the significant relations between neuroimaging parameters and aspects of functional outcome in the whole study sample. Note. Color of the data point refers to the trauma control group (white), mildRF+ TBI group (grey) and moderate/severe TBI group (black). CT = computed tomography; FA = fractional anisotropy; FSIQ = full-scale intelligence quotient. (GIF 57 kb)

High Resolution Image (TIFF 36327 kb)

eFigure 2 Mediation models testing the influence of FA on the relation between moderate/severe TBI and functional outcome. Note. TBI = traumatic brain injury; FSIQ = full-scale intelligence quotient; RAVLT = Rey Auditory Verbal Learning Test; B = raw regression coefficient; SE = standard error. *FA in the cluster of white matter tracts associated with: (1) FSIQ; (2) Digit Span score; and (3) RAVLT Encoding (Fig. 2), respectively. (GIF 35 kb)

High Resolution Image (TIFF 15997 kb)

eTable 1 (DOCX 23 kb)

## Abbreviations

AD: Axial diffusivity
ATR: Anterior thalamic radiation
BCC: Body of corpus callosum
CCB: Cortical part of cingulum bundle
CST: Corticospinal tract
CT: Computed tomography
DTI: Diffusion tensor imaging
FA: Fractional anisotropy
FMa: Forceps major
FMi: Forceps minor
FSIQ: Full-scale IQ
FSL: Functional MRI of the brain software library
GCC: Genu of corpus callosum
GCS: Glasgow coma scale
HCB: Hippocampal part of cingulum bundle
IFOF: Inferior frontal occipital fasciculus
ILF: Inferior longitudinal fasciculus
LOC: Loss of consciousness
MD: Mean diffusivity
MRI: Magnetic resonance imaging
PTA: Post-traumatic amnesia
RAVLT: Rey auditory verbal learning test
RD: Radial diffusivity
RF: Risk factor
SCC: Splenium of corpus callosum
SES: Socio-economic status
SLF: Superior longitudinal fasciculus
TBI: Traumatic brain injury
TBSS: Tract-based spatial statistics
UF: Uncinate fasciculus
WISC-III: Wechsler intelligence scale for children III
