# Supplementary figures and images for: Relevance of neuroimaging for neurocognitive and behavioral outcome after pediatric traumatic brain injury
Source: Brain Imaging Behav. 2017 Jan 14;12(1):29–43. doi: 10.1007/s11682-017-9673-3 (PMC5814510; doi:10.1007/s11682-017-9673-3)

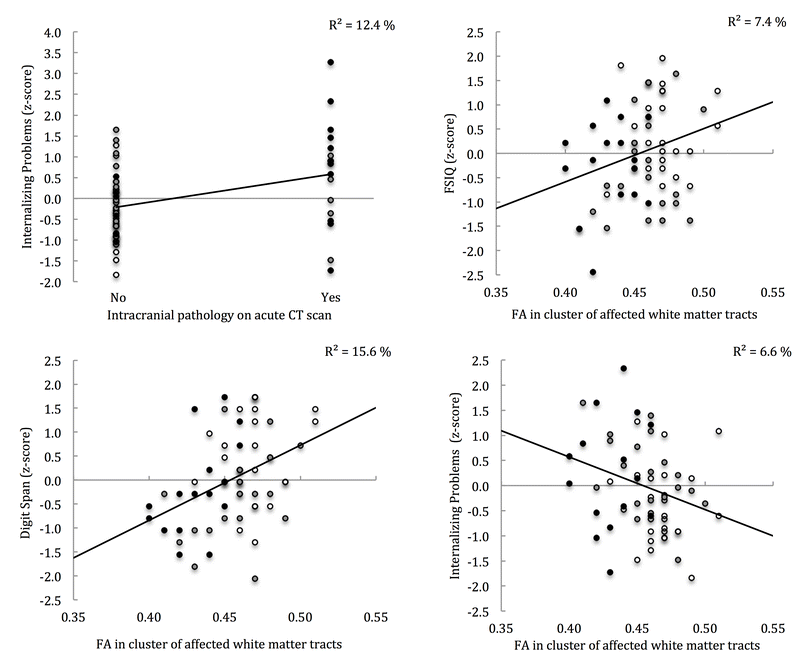

Supplement: Supplementary file 1 — Scatter plots of the significant relations between neuroimaging parameters and aspects of functional outcome in the whole study sample. Note. Color of the data point refers to the trauma control group (white), mildRF+ TBI group (grey) and moderate/severe TBI group (black). CT = computed tomography; FA = fractional anisotropy; FSIQ = full-scale intelligence quotient. (GIF 57 kb) [file 11682_2017_9673_Fig3_ESM.gif]

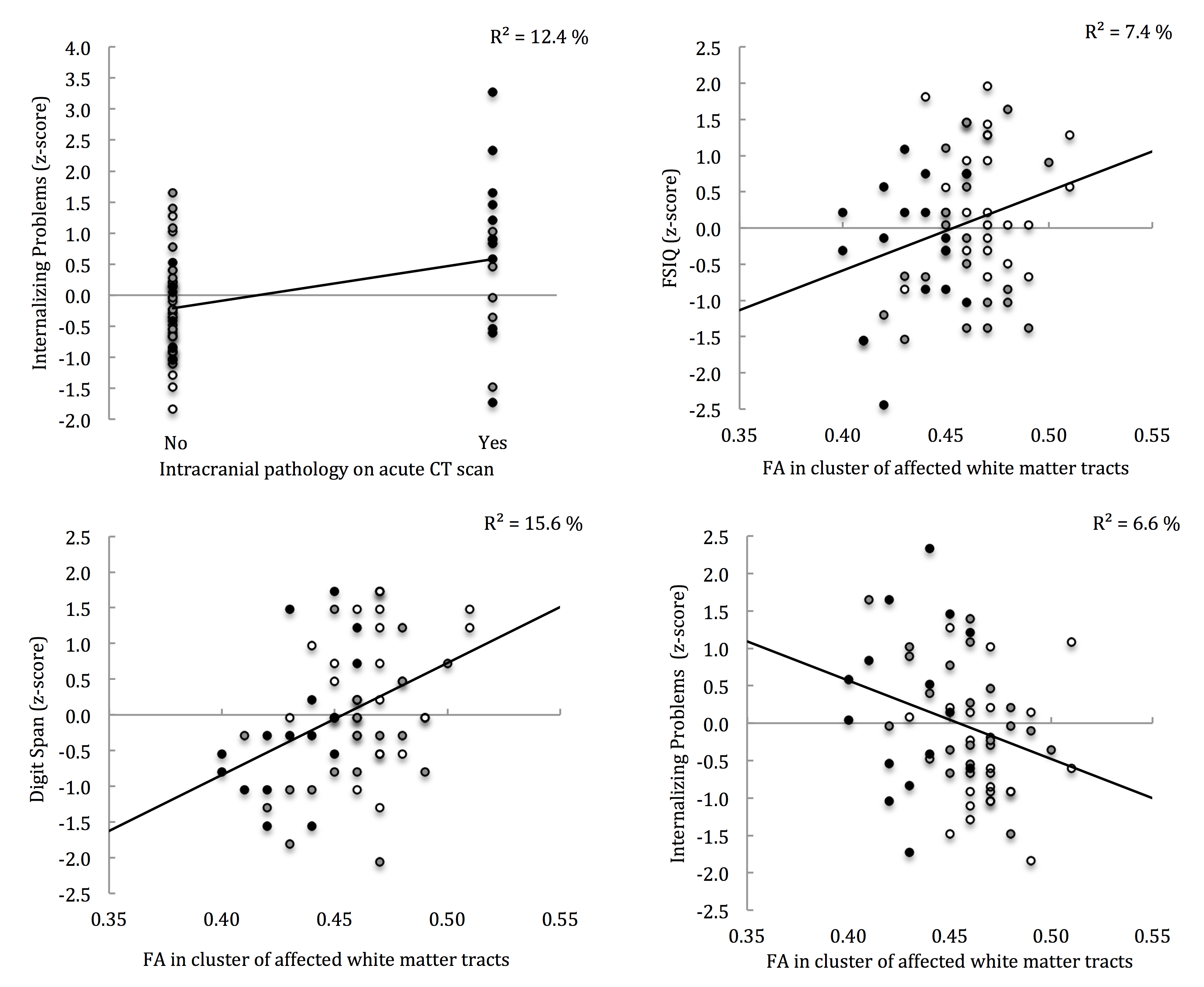

Supplement: Supplementary file 2 — High Resolution Image (TIFF 36327 kb) [file 11682_2017_9673_MOESM1_ESM.tiff]

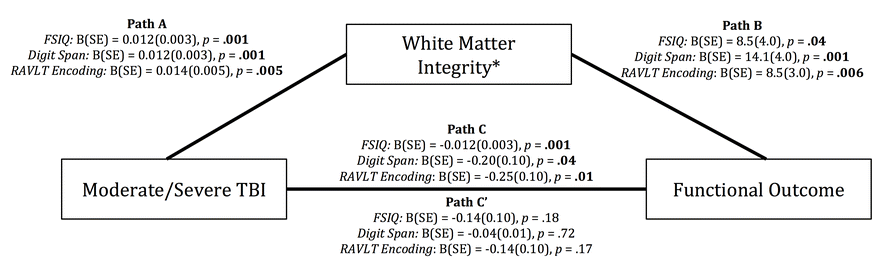

Supplement: Supplementary file 3 — Mediation models testing the influence of FA on the relation between moderate/severe TBI and functional outcome. Note. TBI = traumatic brain injury; FSIQ = full-scale intelligence quotient; RAVLT = Rey Auditory Verbal Learning Test; B = raw regression coefficient; SE = standard error. *FA in the cluster of white matter tracts associated with: (1) FSIQ; (2) Digit Span score; and (3) RAVLT Encoding (Fig. 2), respectively. (GIF 35 kb) [file 11682_2017_9673_Fig4_ESM.gif]

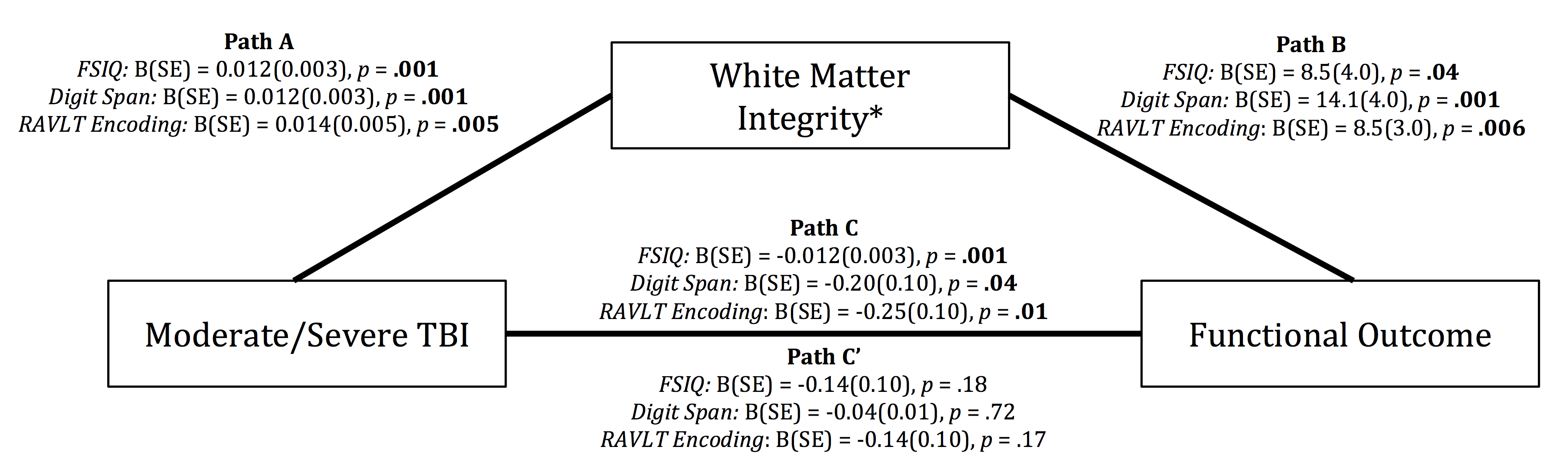

Supplement: Supplementary file 4 — High Resolution Image (TIFF 15997 kb) [file 11682_2017_9673_MOESM2_ESM.tiff]
