# Supplementary material for: Relevance of neuroimaging for neurocognitive and behavioral outcome after pediatric traumatic brain injury
Source: Brain Imaging Behav. 2017 Jan 14;12(1):29–43. doi: 10.1007/s11682-017-9673-3 (PMC5814510; doi:10.1007/s11682-017-9673-3)
Supplement: Supplementary file 5 — (DOCX 23 kb) [file 11682_2017_9673_MOESM3_ESM.docx]

Page 2 eTable 1. Findings on acute scans of children with intracranial pathology.

eTable 1. Findings on acute CT-scans of children with intracranial pathology after TBI.

| Group | Acute CT-scan |
| --- | --- |
| *Mild^RF+^ TBI* |  |
| Patient 1 | Epidural hematoma (right occipital) |
| Patient 2 | Epidural hematoma (right frontobasal) |
| Patient 3 | Epidural hematomas (temporal & orbital) |
| Patient 4 | Epidural hematoma (right frontal) |
| Patient 5 | Epidural hematoma (temporal) |
| Patient 6 | Subdural/epidural hematoma (left occipital) |
| *Moderate/Severe TBI* |  |
| Patient 7 | Probable small contusion of cerebellar peduncles |
| Patient 8 | Intraventricular hemorrhage |
| Patient 9 | Cerebral contusion (left frontal) |
| Patient 10 | Subarachnoid hemorrhage (left frontal and right parietal) |
| Patient 11 | Subdural hematoma (right frontal) |
| Patient 12 | Hemorrhagic cerebral contusions (left frontal) |
| Patient 13 | Subdural hematoma (right frontoparietal) |
| Patient 14 | Epidural hematomas (left cerebellar, right temporal, left temporolateral and left temporobasal) |
| Patient 15 | Subdural hematoma (left temporal) |
| Patient 16 | Epidural hematoma (frontoparietal) |
| Patient 17 | Probable subarachnoid hemorrhage (tentorium) |

*Note.* RF = risk factor; TBI = traumatic brain injury.
